# Four years monitoring of the endangered European plethodontid salamanders

**DOI:** 10.1038/s41597-024-03555-y

**Published:** 2024-06-27

**Authors:** Luca Coppari, Milos Di Gregorio, Claudia Corti, Stefano Merilli, Manuela Mulargia, Roberto Cogoni, Raoul Manenti, Gentile Francesco Ficetola, Enrico Lunghi

**Affiliations:** 1 Department of Life, Health and Environmental Sciences, University of L’Aquila, L’Aquila, Italy; 2 Department of Veterinary Science, University of Pisa, Pisa, Italy; 3 Natural History Museum of the University of Firenze, Museo “La Specola”, Firenze, Italy; 4 Speleo Club Firenze, Firenze, Italy; 5 Via Isalle 4, Siniscola, Italy; 6 Unione Speleologica Cagliaritana, Quartu Sant’Elena, Cagliari, Italy; 7 Department of Environmental Science and Policy, University of Milano, Milano, Italy; 8 Laboratoire d’Écologie Alpine (LECA), University of Grenoble Alpes, CNRS, Grenoble, France; 9 Natural Oasis, Prato, Italy; 10 Unione Speleologica Calenzano, Calenzano (Florence), Italy

**Keywords:** Herpetology, Biodiversity

## Abstract

The ongoing biodiversity crisis is strongly threatening amphibians, mostly because of their peculiar physiology, their sensitivity to climate change and the spread of diseases. Effective monitoring involving assessments of pressure effects across time and estimation of population trends play a key role in mitigating amphibian decline. To improve implementation of standardized protocols and conservation efforts, we present here a dataset related to one of the amphibian genera whose onservation status is considered the most declining according to the IUCN. We report information on 66 populations of the endangered European cave salamanders, genus *Speleomantes*, that was collected through a standardized monitoring along a four-year period (2021–2024). Demographics data of the populations and fitness-related data of single individuals are reported. Furthermore, we include 3,836 high quality images of individuals that can allow to perform studies aiming to assess the phenotypic variability within the genus, and to perform long-term capture-mark-recaptured studies.

## Background & Summary

Biodiversity crisis is a major ongoing problem occurring at the global scale, spanning all types of environments^1–3^. Such crisis is directly or indirectly enhanced by multiple human activities that determine, or favour, the conditions that pose species at risk of extinction^4^. Among them, environmental changes, and the introduction of new species (either competitors or pathogens) have substantial impacts. In the first case, the relatively fast environmental changes, for example caused by climate change or by pollutants, can quickly create conditions that overcome species physiological limits, preventing their potential gradual adaptation to the novel environmental conditions and driving populations to a dramatic end^5–8^. In the second case, native species have to deal with newly introduced species that may be stronger competitors^9,10^ or that can overcome immunological barriers of individuals^11,12^. All these processes often result in the progressive disappearance of local biodiversity.

Amphibians are the most threatened vertebrates worldwide^11,13^. These vertebrates are generally characterized by a series of specific traits that synergistically enhance their sensitivity to human impacts. For example, many amphibians are characterized by a biphasic life cycle, and their life history stages require both aquatic and terrestrial environments. That means that disturbance in just one of the two environments can provoke significant effects that can lead to the local extinction of the species. Further, amphibians completely rely on the environment for their physiological homeostasis, for example to maintain the body temperature within their optimal range or to balance the water loss^14–16^. Considering that most of amphibians are able to exploit a narrow range of environmental conditions^17,18^, even a small change in such conditions can be unbearable. Furthermore, amphibians are often characterized by a low dispersal ability^19^, a characteristic that hampers species to track their preferred conditions, thus exacerbating the impacts of environmental changes.

Long-term monitoring is a necessary prerequisite to set effective protection of amphibians and prevent their declines and extinctions^20^. Repeated surveys are indeed crucial for early identification of population decline and the emergence of potential threats^21^, allowing timely actions and mitigations of related harmful effects^22,23^. With this dataset we provide data from repeated surveys performed on the strictly protected *Speleomantes* cave salamanders, the only plethodontid species present in Europe^24,25^. There are eight allopatric species of *Speleomantes*, endemic to specific areas distributed in mainland Italy, in the Republic of San Marino, in south-eastern France and on the island of Sardinia^24,26^. Populations of natural hybrids (between *S. ambrosii* and *S. bianchii*) are also known from a small contact zone located in Apuan Alps (Northern Tuscany)^27^. Their overall conservation status is alarming: one species is Near Threatened, one Vulnerable, four Endangered and two Critically Endangered (www.iucnredlist.org; last access on 05 February 2024). A mandatory monitoring activity of protected species has been imposed by the European Union (Article 17 of the Habitats Directive; https://nature-art17.eionet.europa.eu/article17/) with the aim of constantly updating their conservation status; however, very limited data is currently available for the genus *Speleomantes*. In addition to providing a large amount of data covering a four-year period, this dataset offers the possibility of being combined with those previously published^28–30^, allowing the monitoring period for some populations to be extended to seven years. The availability of standardized data from multiple conspecific populations can also increase the possibility of detecting intraspecific variation as key traits. This can contribute to the conservation of not just species, but also of populations and intraspecific diversity^31–33^

## Methods

The monitoring activity involved multiple surveys on 66 populations of *Speleomantes* salamanders in the period 2021–2024 (Tables 1 and 2). The surveyed populations were subterranean or fully epigean. In the first case, the subterranean environments (both natural and artificial) were surveyed entirely or up to the point where the exploration required the use of speleological equipment; in surface environments, specific plots delimited the study area. The surveys were carried out producing an average sampling effort of approximately 27 m^2^/minute^34^. *Speleomantes* were opportunistically searched and captured within the sampling area: these were the active individuals and those spotted hiding under stones or inside crevices. All captured individuals from a population were temporarily placed in disinfected (using bleach) fauna boxes until processing. A portable photo studio has been set in the field which allows high quality images to be obtained from which it is possible to extrapolate biometric data^35,36^. This portable studio is designed to obtain high-quality images by controlling the position of the camera and lights (i.e. flash), allowing individuals to be shot perpendicularly from above and producing comparable images useful for individual recognition through the use of the dorsal pattern^37^. This also allows a high standardization of the coloration of salamanders^35^. During each survey, we shot a first image taken on a Pantone Calibrite ColorChecker Passport Photo 2 (for brevity, hereafter Pantone); this allowed us to obtain the white reference for image calibration, and the length reference to insert within each image a as a unit of measurement. Salamanders were weighed on a digital scale (0.01 g) and visually inspected to record multiple data.

**Table 1:** Summary of the surveys carried out in the period 2021–2024.

Species	Population_code	Year	Month	N pictures
*S. ambrosii bianchii*	S_ambrosii_bianchii1	2021	July	103
		2023	October	49
*S. ambrosii ambrosii*	S_ambrosii2	2023	June	117
	S_ambrosii3	2023	June	39
	S_ambrosii4	2023	June	41
*Speleomantes* hybrid	S_hybrid1	2023	June	26
	S_hybrid2	2023	June	37
	S_hybrid3	2023	June	17
	S_hybrid4	2021	July	44
		2023	June	15
	S_hybrid5	2021	June	11
		2023	June	8
		2023	September	11
	S_hybrid7	2023	October	123
	S_hybrid8	2023	June	43
	S_hybrid9	2023	June	10
*S. italicus*	S_italicus1	2022	October	10
		2023	September	7
	S_italicus2	2022	October	35
	S_italicus3	2022	October	38
	S_italicus4	2023	May	31
	S_italicus6	2023	September	2
	S_italicus7	2023	September	12
	S_italicus8	2022	November	185
		2023	October	153
	S_italicus9	2021	June	29
		2023	September	60
	S_italicus10	2021	July	97
	S_italicus11	2022	October	11
		2022	November	28
		2023	October	36
	S_italicus12	2022	October	42
		2023	October	57
	S_italicus13	2022	November	24
	S_italicus14	2022	November	63
		2023	April	22
	S_italicus15	2023	April	13
	S_italicus16	2023	April	109
	S_italicus17	2023	May	10
		2023	October	33
	S_italicus18	2023	November	35
*S. strinatii*	S_strinatii1	2023	October	11
	S_strinatii2	2023	June	16
	S_strinatii5	2023	June	59
	S_strinatii8	2021	June	16
	S_strinatii9	2021	June	87
	S_strinatii11	2021	June	45
	S_strinatii12	2022	November	65
	S_strinatii13	2021	June	23
	S_strinatii14	2023	June	24
	S_strinatii15	2021	June	68

For each mainland species (including hybrids), the number of populations surveyed, the periods in which surveys were conducted and the total number of individuals photographed are reported.

**Fig. 1:**
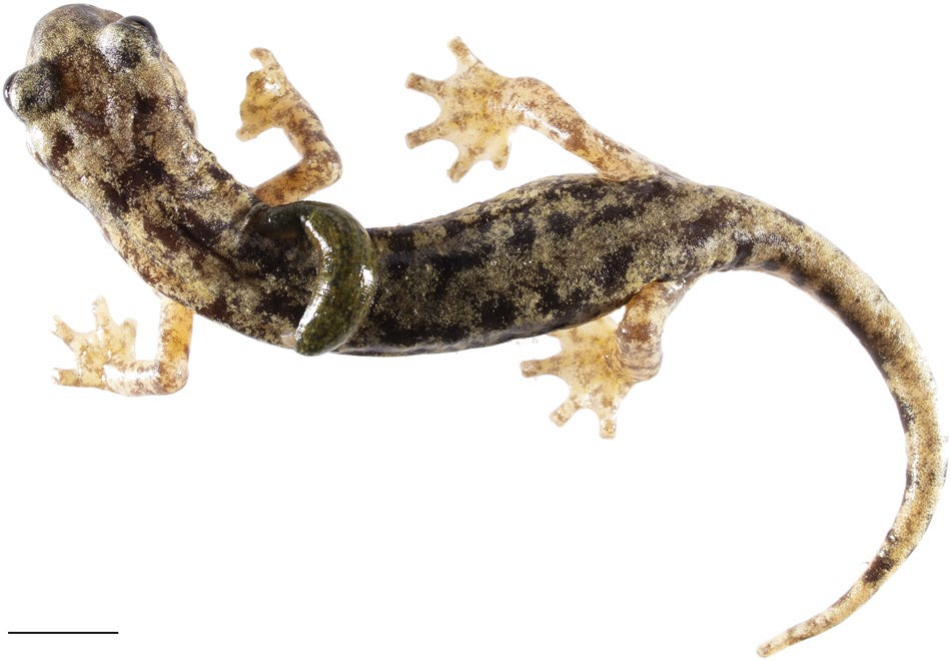
Example of the high-quality images composing the dataset. In this picture an individual of *Speleomantes flavus* is parasitized by a leech of the genus *Batracobdella*. Scale bar = 10 mm.

**Fig. 2:**
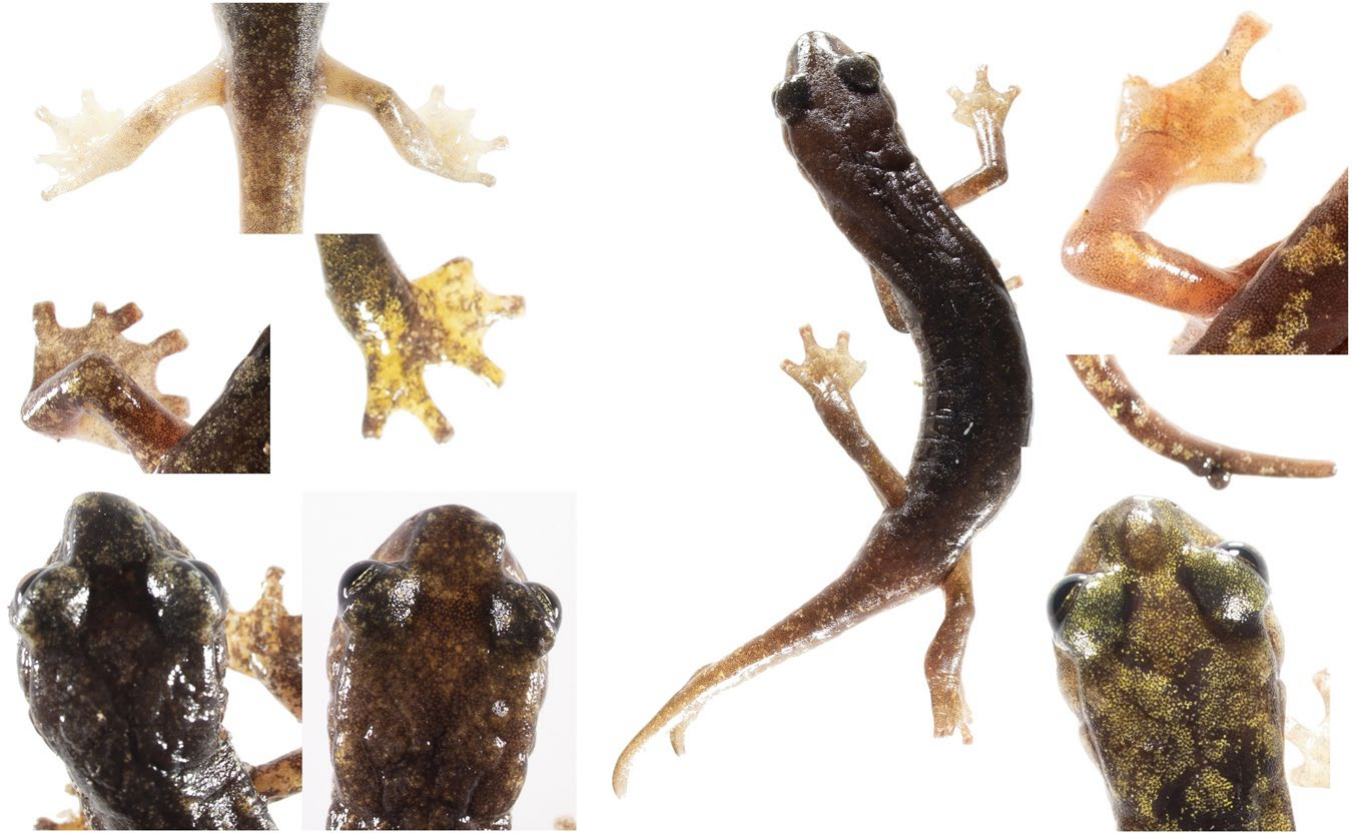
Some examples of malformations observed in *Speleomantes* during our study.

An initial inspection was carried out to assess the presence of ectoparasites, i.e. leeches of the genus *Batracobdella*, which are known to parasitise the Sardinian *Speleomantes*^24,38^ (Fig. 1). When leeches were present, they were counted and weighed separately. Subsequently, a check was carried out for any malformation (e.g., forked tail, foot shape^39,40^;) (Fig. 2). In some of the monitored populations, individuals were previously marked using either the Visual Implant Elastomers^41^ and Visual Implant Alpha tags^42^, we used an UV light to assess the presence of these tags. Tags were placed on arms, flanks and the base of the tail (for further information on tag implantation refer to^41,42^). The individuals’ head was then checked to assess the presence of the “mental gland”, a male sexual character located under the lower jaw^24^; all salamanders found with this character were considered adult males. Considering the exclusivity of males in showing distinctive morphological characters, adult females and juveniles are generally distinguished based on body size^24^. *Speleomantes* can lose and regrow their tail to avoid predation^43^; this makes total length (TL) unreliable for making this distinction. We then used the snout-vent length (SVL, in mm) to size individuals and distinguish between juveniles and adult females^44^. The threshold was set based on the average minimum size observed for gravid females and mature males^45,46^: for the three continental species (*S. strinatii*, *S. ambrosii* and *S. italicus*), hybrids and for *S. genei* it was set at 50 mm, while for the other “larger” Sardinian species (*S. flavus*, *S. supramontis*, *S. imperialis* and *S. sarrabusensis*) to 55 mm. This distinction was only possible in the laboratory after estimating the SVL of individuals from the images (see below).

Camera RAW files (.CR2) were opened and processed with Adobe Photoshop. For each population we first opened the Pantone photo with Adobe Camera Raw and set white calibration using the *White Balance Tool* function; the resulting profile was used to white balance all photos taken during a single session. We then set the scale to 10 mm using the Pantone reference measurement; the same amount of pixels was used for the entire population thanks to the fixed position of the camera^35^. The images were cropped and cleaned by adding a white background. After adding the reference scale they were transformed into JPEG, allowing to reduce the weight of the file (~2–3 MB) without compromising its quality^28^ (Fig. 1). We then used Fiji^47^ to estimate individuals SVL^36^. After setting the image scale (*straight line*) via the scale bar, we used the *segmented line* to draw a line from the snout to the tip of the tail, following the centre of the body; this allowed to measure the individual’s total length. Another segmented line was drawn from the tip of the snout to the extremity of the body, that is the point that corresponds to the opening of the cloaca^36^; this allowed us to estimate SVL. The differences between TL and SVL were used to obtain tail length.

## Data Records

The dataset presented here (*Speleomantes* photographic dataset 2021–2024, available on *figshare*^48^) consists of 3,836 high-quality images of individuals from 66 *Speleomantes* populations, including the eight known species distributed in mainland Italy (including the Republic of San Marino) and Sardinia^24^, as well as hybrid populations occurring in the north (i.e., natural populations) and south (i.e., introduced populations) of Tuscany^27,49^. Detailed information on *Speleomantes* populations (coded following^28^) is shown in Tables 1 and 2.

**Table 2:** Summary of the surveys carried out in the period 2021–2024.

Species	Population_code	Year	Month	N pictures
*S. flavus*	S_flavus1	2024	January	10
	S_flavus3	2022	June	69
		2022	October	66
		2023	September	63
	S_flavus4	2022	June	8
		2022	October	12
		2023	September	10
	S_flavus6	2022	June	20
	S_flavus7	2022	June	4
		2023	September	13
*S. genei*	S_genei1	2022	June	22
		2022	October	9
		2023	September	24
	S_genei2	2022	June	67
		2022	October	93
		2023	September	67
	S_genei4	2022	March	23
		2022	October	11
	S_genei4_1	2024	January	20
	S_genei5	2023	September	58
	S_genei6	2024	January	16
	S_genei7	2024	January	28
	S_genei8	2024	January	11
	S_genei9	2024	January	23
*S. imperialis*	S_imperialis1	2022	June	48
		2022	October	17
		2023	September	38
	S_imperialis2	2022	June	16
		2022	October	10
		2023	September	8
	S_imperialis3	2022	June	74
		2022	October	58
		2023	September	109
	S_imperialis4	2023	September	26
	S_imperialis5	2024	January	4
	S_imperialis6	2024	January	22
	S_imperialis7	2024	January	10
*S. sarrabusensis*	S_sarrabusensis1	2022	June	8
		2023	September	12
	S_sarrabusensis2	2022	June	6
		2023	September	4
	S_sarrabusensis4	2022	March	39
		2024	January	25
*S. supramontis*	S_supramontis1	2022	June	41
		2022	October	85
		2023	September	15
	S_supramontis2	2022	June	43
		2022	October	50
		2023	September	41
	S_supramontis3	2022	June	11
		2023	September	19

For each Sardinian species, the number of populations surveyed, the periods in which surveys were conducted and the total number of individuals photographed are reported.

Each image comes with additional information about the individual’s biometric data (e.g., size, weight) and condition (e.g., presence of parasites, malformations) (Information on *Speleomantes* individuals^48^). The .CSV supporting dataset is composed as shown in Table 3.

**Table 3:** Description of the accompanying dataset “Information on *Speleomantes* individuals”.

Column name	Data description
ID	Progressive record ID
Domain	Subterranean vs Surface
Habitat	Cave (terms including all types of subterranean environments), Forest, Garigue/Scree, Stone wall
Latitude and Longitude	Coordinates of the sampled site. Precision is reduced for conservation purpose^61^
Elevation	Meters above sea level
Genus and Species	Scientific name of the species
Population code	Population identifier
Country, Region, Province	Geographic reference of the sampled population
Month, Year	Identifies the period of the survey
Area	The surveyed area (m^2^)
Tag	Unique code identifying marked individuals with Alpha Tag or Visual Implant Elastomers. For the latter, the code is composed by: a letter indicating the colour (b = blue, g = green, p = pink, y = yellow) and a series of number indicating the specific body part (see^41^ for further information)
Sex	M = adult male; F = adult female; J = juvenile
Eggs	Indicates whether developed eggs were visible from salamanders’ abdomen
Tail issue	Indicates whether the tail shows normal (0) or shortened (1) length
Leech number and weight	Indicates how many leeches were parasitizing the salamander and the overall weight of the parasites
Malformation	Indicates the presence of a body malformation. This could be related to limb, head, tail. NA = no malformations observed
Weight	Individual weight (g)
SVL	Snout-vent length (mm)
Tail	Tail length (mm)
TL	Total length (mm)
Picture number	Identifies the image of the individuals photographed during each survey
Scale bar	Indicates the length (mm) of the scale bar in images
N_operators	Indicates the number of researchers involved in salamanders searching activity

This Excel table contains qualitative information of each photo composing the main dataset.

## Technical Validation

**Fig. 3:**
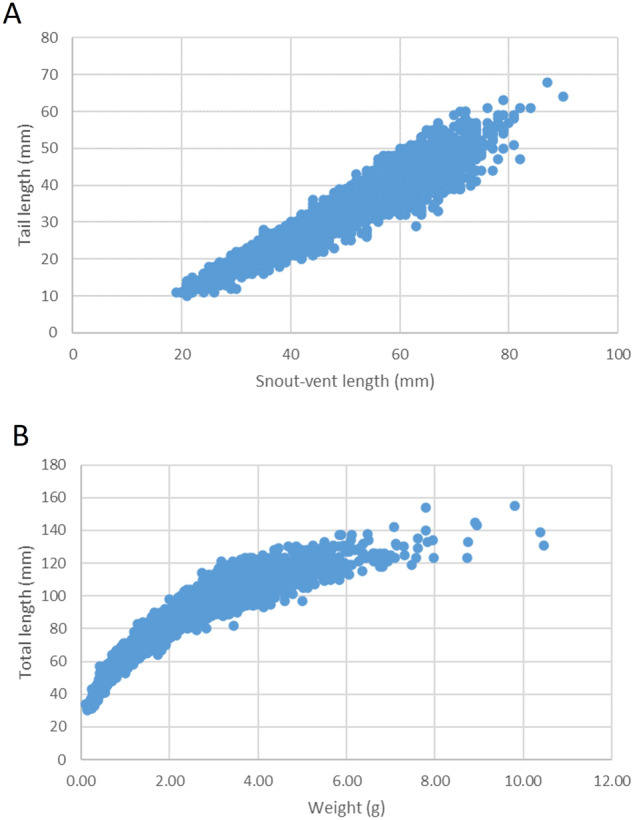
Linear regression plots used to evaluate the presence of erroneous data. (**A**) Correlation between SVL and tail length; (**B**) correlation between TL and weight.

Surveys were performed during a single day and by operators adopting the same sampling effort. The standardisation and quality of images provided here is guaranteed by the adoption of the best protocol described for these species^28,29^. The snout-vent and total length were estimated through analysis of the high-quality images provided here; this methodology allows reliable estimates with very small measurement error (~2 mm) even if measures are taken by a non-expert operator^36^. Potential errors due to measurement or transcription were double checked by plotting the data (SVL vs TL and TL vs weight) (Fig. 3). Anomalous data were compared to paper field data, and dubious measurements were repeated. Considering the SVL threshold used to distinguish juveniles (50 and 55 mm for normal and giant species, respectively), all individuals with SVL lower than the respective threshold were considered juveniles.

## Usage Notes

This dataset has broad application. In addition to being an important tool for estimating and monitoring the abundance of populations over time, and for studying demographic parameters (e.g., age distribution, survival, growth rate) as specified above, we list some other important uses here. The conditions of individuals can be evaluated from different points of view: in addition to verifying the presence of ectoparasites and malformations, using the ratio between total length (TL) and weight we can evaluate the body condition of each individual^50^. This fitness-related trait allows inter- and intrapopulation comparisons in space and time^51,52^, making it possible to evaluate factors that negatively influence individual condition^53^. In this circumstance, the use of TL in estimating individual body condition is more appropriate, since these salamanders usually accumulate fat in the tail^54^. The images can be used for geometric morphometry^55^, for coloration analyses^47,56,57^, and can also be used to test the reliability of different software for the automatic photographic identification of individuals^58–60^.

## Data Availability

No code was used in this study.
